# Ionic liquid-assisted formation of cellulose/calcium phosphate hybrid materials

**DOI:** 10.3762/bjnano.5.167

**Published:** 2014-09-16

**Authors:** Ahmed Salama, Mike Neumann, Christina Günter, Andreas Taubert

**Affiliations:** 1 Institute of Chemistry, University of Potsdam, D-14476 Potsdam, Germany; 2 Cellulose and Paper Department, National Research Center, El-Tahrir Street, Dokki, Cairo, Egypt; 3 Institute of Earth and Environmental Sciences, University of Potsdam, D-14476 Potsdam, Germany

**Keywords:** biomineralization, calcium phosphate, carbohydrates, cellulose, hybrid materials, ionic liquid

## Abstract

Cellulose/calcium phosphate hybrid materials were synthesized via an ionic liquid-assisted route. Scanning electron microscopy, transmission electron microscopy, energy-dispersive X-ray spectroscopy, X-ray diffraction, infrared spectroscopy, and thermogravimetric analysis/differential thermal analysis show that, depending on the reaction conditions, cellulose/hydroxyapatite, cellulose/chlorapatite, or cellulose/monetite composites form. Preliminary studies with MC3T3-E1 pre-osteoblasts show that the cells proliferate on the hybrid materials suggesting that the ionic liquid-based process yields materials that are potentially useful as scaffolds for regenerative therapies.

## Introduction

One of the key advantages of carbohydrates, especially cellulose and chitin, is their abundance and favorable properties such as mechanical robustness and biocompatibility [1–4]. Moreover, the growth (mineralization) of calcium phosphate on polysaccharides may lead to composites with properties that are useful for the regeneration of hard tissue even though the chemical composition of these materials is different from the original biomaterial [5–11]. Unfortunately, the synthesis of carbohydrate-based hybrid materials is not straightforward. This is due to the fact that many carbohydrates exhibit low solubilities in aqueous media. Aqueous solutions, however, are the most commonly used media for calcium phosphate mineralization [12–13]. As a result, mineralization of carbohydrates often yields heterogeneous materials with properties that are not suited for an application. In spite of this, a number of authors have reported the successful mineralization of carbohydrates with various calcium phosphates.

Falini and coworkers used β-chitin from a squid pen for mineralization of octacalcium phosphate (OCP) and hydroxyapatite (HAP) [14–15]. They found a distinct change of the chitin fiber organization on OCP mineralization. Moreover, the OCP–HAP transition is delayed with respect to OCP grown in the absence of the carbohydrate matrix. One of the issues of chitin, however, is again its limited solubility in most mineralization media. This limits the processing and mineralization efficiencies. Chitosan, which exhibits a higher water solubility than chitin, has therefore been used as an alternative scaffold for calcium phosphate mineralization [16–17]. Among others, chitosan/HAP scaffolds show good osteoconductivity and biodegradability, as has been shown for some synthetic composites in rats [18].

Chiono et al. developed a photochemical approach towards the triggered nucleation of calcium phosphate on chitosan cast films [19]. Mineralization is induced by photoexcitation of fluorescein molecules grafted to the chitosan films. The authors claim that the formation of local positive charges by electron transfer from the fluorophore to reactive species in the surrounding medium like O_2_ or water leads to singlet oxygen radicals and superoxide radical anions. According to the authors, these may then act as nucleation sites. One unresolved question here is the fact that these results differ significantly from other work [16,18] where calcium phosphate deposition on chitosan was equally successful, but without the need to photoactivate the mineralization reaction.

Besides chitin and chitosan, carboxymethyl inulin (CMI) [20–21] and carboxymethyl cellulose (CMC) [22–23] have been studied as mineralization additives. Composites of CMC, calcium phosphate nanoparticles, and the antibiotic chlorhexidine efficiently remineralize dentin tubules [23]. In contrast, CMI inhibits or at least delays calcium phosphate mineralization [20–22].

There are also a few reports on the mineralization of unmodified cellulose [11,24–33], but like in the case of chitin, the poor solubility of cellulose in conventional solvents hampers the development of true calcium phosphate/carbohydrate hybrid materials because it prevents, or at least dramatically reduces, the penetration of the precursor ions into the carbohydrate templates and thus results in materials mostly exhibiting surface or near-surface mineral layers.

The most straightforward strategy towards real, nanostructured and hierarchical carbohydrate/calcium phosphate composites would therefore be a synthesis protocol using a solvent that is able to dissolve carbohydrates as single molecules or very small aggregates. At the same time the solvent should enable the growth of calcium phosphate.

Ionic liquids (ILs) could provide a viable access for the synthesis of such nanoscale carbohydrate/inorganic hybrids. Some ILs dissolve up to 25 wt % of cellulose [34–37]. This efficiency has mainly been attributed to the ability of the ILs to break hydrogen bonds, which is the key interaction stabilizing cellulose and chitin [34,38–40]. Moreover, ILs are efficient reaction media for the synthesis of new and interesting inorganic materials [35,41–45] although there are only a few reports on IL-based protocols for the synthesis of carbohydrate/inorganic hybrid materials.

Mumalo-Djokic et al. studied the formation of ZnO/carbohydrate hybrid materials using a hydrated IL, tetrabutylammonium hydroxide [TBA][OH], as the solvent and hydroxide source for ZnO formation [46]. This study revealed significant differences between the two carbohydrates studied, cellulose and starch. While starch was soluble in the water/IL mixture, cellulose was, due to the high water content in the reaction mixture, not. As a result, while the mineralization of starch led to a nanoscale hybrid material, the mineralization of cellulose led to cellulose fibers with a high degree of surface mineralization. In spite of this, the cellulose fibers appeared to “imprint” some features of their surface structure on the mineral layers.

Venkataramanan et al. synthesized cellulose/TiO_2_ hybrids via a sol–gel reaction in 1-butyl-3-methylimidazolium chloride, [Bmim][Cl] [47]. Ti(OBu)_4_ was used as TiO_2_ precursor and a network of TiO_2_ layered fibers was observed after the sol–gel reaction. Amarasekara and Owereh prepared cellulose carbamate/silica hybrid materials in [Bmim][Cl] [48]. Cellulose-based hybrid materials with calcium carbonate [49], copper oxide [50], or calcium silicate [51] have been grown in [Bmim][Cl]. Finally, there is a report on the synthesis of cellulose/calcium phosphate composites using ILs [52]. The authors of this study, however, did not grow inorganic matter in the IL, but dispersed prefabricated hydroxyapatite (HAP) nanoparticles into a solution of cellulose in [Bmim][Cl] to form composites with limited homogeneity.

Besides the approaches introduced above, [Bmim][Cl] has also been used for calcium carbonate precipitation [53]. [Bmim][Cl] is thus a prime candidate for the generation of new calcium phosphate/carbohydrate hybrid materials. The current study therefore evaluates the potential of [Bmim][Cl] for the synthesis of well-defined calcium phosphate/cellulose composites with a defined morphology, chemical composition, calcium phosphate crystal phase, crystal organization, and suitable compatibility for cells. The approach is based on the precipitation of calcium phosphate from IL/cellulose solutions rather than adding pre-fabricated calcium phosphate nanoparticles to the IL/cellulose solution and thus provides a rather simple, one-step approach towards cellulose/calcium phosphate hybrid materials.

## Experimental

**Materials.** [Bmim][Cl] (≥95%, Aldrich) was freeze-dried from water. The final water content was below 0.3%, as determined by volumetric Karl Fischer titration. After freeze-drying the IL was stored under argon until use. Microcrystalline cellulose (extra pure, average particle size 90 µm, Acros), calcium chloride dihydrate CaCl_2_·2H_2_O (extra pure, Merck), dibasic potassium phosphate K_2_HPO_4_ (≥98%, Sigma-Aldrich), sodium dihydrogen phosphate dihydrate NaH_2_PO_4_·2H_2_O (≥98%, Roth), and glacial acetic acid (100%, p.a., water content below 0.1%, Roth), NaOH (puriss. p.a. ACS, pellets, ≥98%, Sigma-Aldrich), and ethanol (p.a., absolute, Merck) were used as received.

**Calcium phosphate synthesis.** 0.6 g of powdered calcium chloride (4.1 mmol) were dissolved in 6 g of [Bmim][Cl] at 80 °C under vigorous stirring. After complete dissolution, 2.46 mmol of the phosphate precursor (0.43 g of dibasic potassium phosphate or 0.38 g of sodium phosphate) were added at 80 °C, yielding a reaction mixture with a Ca/P ratio of 1.67. Then 0.4 mL of ethanolic NaOH or glacial acetic acid was added and the ethanol was removed by evaporation. The reaction mixture was subsequently stirred for 24 or 48 h at 80 °C. The reaction products were precipitated by adding an excess amount of water to the reaction mixture after cooling. The precipitates were filtered, washed with distilled water, and the IL was removed from the products via Soxleth extraction (methanol, 48 h). The purified products were dried at 40 °C for 24 h in a vacuum oven. Samples are labeled CP^Xy^, where X = NaOH or GAA (glacial acetic acid) indicates the additive and y = 24 or 48 indicates the reaction time, 24 or 48 h. For example, CP^NaOH24^ is a sample grown in the presence of NaOH for 24 h.

**Preparation of cellulose/calcium phosphate hybrid materials.** Cellulose was dissolved in [Bmim][Cl] at 80 °C overnight in different weight fractions (Table 1). 0.6 g of powdered calcium chloride (4.1 mmol) per 6 g of IL was added to the cellulose/IL solution at 80 °C under vigorous stirring. After complete dissolution/dispersion, 2.46 mmol of the phosphate precursor (0.43 g of dibasic potassium phosphate or 0.38 g of sodium phosphate) were added at 80 °C, yielding a reaction mixture with a Ca/P ratio of 1.67. Then 30 µL GAA or ethanolic NaOH were added and the ethanol and water from the inorganic precursor salts were removed under high vacuum (10^−3^ mbar) for 30 min. The reaction mixture was subsequently stirred for 24 or 48 h at 80 °C during which time a white precipitate formed. The reaction products were precipitated by adding an excess amount of water to the reaction mixture after cooling. The precipitate was filtered, washed with distilled water, and the IL was extracted from the products by Soxleth extraction with methanol for two days. The products were subsequently dried at 40 °C for 24 h in a vacuum oven.

**Table 1:** Samples investigated in this study. CP is calcium phosphate, GAA is glacial acetic acid, 24 and 48 are reaction times in hours.

Sample	Phosphate precursor^a^	GAA or NaOH	Reaction conditions

CP^GAA24^	NaH_2_PO_4_·2H_2_O	GAA	24 h, 80 °C
CP^GAA48^	NaH_2_PO_4_·2H_2_O	GAA	48 h, 80 °C
CP^NaOH24^	K_2_HPO_4_	NaOH	24 h, 80 °C
CP^NaOH48^	K_2_HPO_4_	NaOH	48 h, 80 °C

^a^Calcium precursor was always CaCl_2_·2H_2_O.

**Characterization.** Attenuated total reflection-Fourier transform infrared spectroscopy was done on a Thermo Nicolet FT-IR Nexus 470 with a diamond crystal. Spectra were recorded from 500 to 4000 cm^−1^ with a resolution of 2 cm^−1^. X-ray diffraction patterns were recorded with a Siemens D5005 (Cu Kα, 0.154 nm) between 3 and 70° 2θ with a step size of 0.02° per second. Samples were mounted on a silicon support. Scanning electron microscopy was done on a FEI Phenom operated at 5 kV. Transmission electron microscopy was done on a Zeiss 912 Omega operated at 120 kV. Cross sections were obtained with a Leica Ultra Cut Microtome. For sectioning, the powder samples were embedded in “LR white” resin (Plano GmbH). Samples were cut at 1 mm/s at room temperature. Energy dispersive X-ray spectroscopy was done on a JEOL JSM 6510 SEM with tungsten hairpin filament (15 kV) and an Oxford INCAx-act SN detector with a resolution of 135 eV at 5.9 keV. Elemental analysis was done on a Vario EL III analyzer. Thermogravimetric analysis/differential thermal analysis was done on a Linseis STA PT-1600 thermal balance in air from 20 to 600 °C with a heating rate of 10 K/min and air flow of 50 mL/min.

## Results

### Calcium phosphate precipitated without cellulose

**Figure 1:**
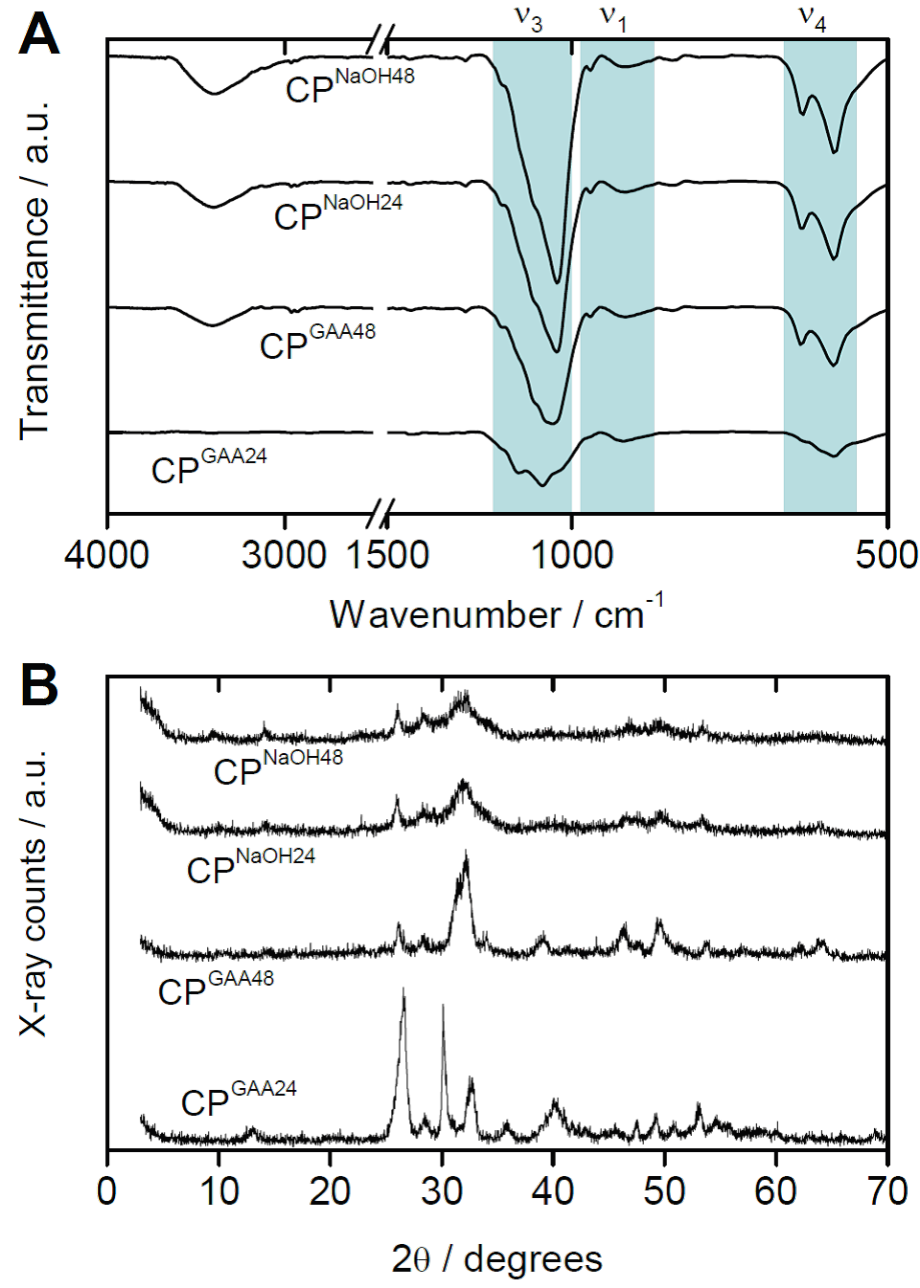
(A) ATR-FTIR spectra and (B) XRD patterns of calcium phosphates obtained from [Bmim][Cl]. The reflection at 14.2 degrees 2θ is from the sample holder (Si).

Table 1 summarizes the samples obtained after reaction in [Bmim][Cl]. Figure 1 shows the XRD patterns and the FTIR spectra of the reaction products after purification. The IR spectra of the calcium phosphates obtained by reaction in the presence of glacial acetic acid (GAA) after 24 h (CP^GAA24^, for details of labeling see Experimental part) show no band in the range of 3500 cm^−1^, which suggests that these precipitates are relatively free from water or hydroxy groups. In contrast, all other samples (CP^GAA48^, CP^NaOH24^, CP^NaOH48^) show strong bands at 3350 to 3360 cm^−1^ indicating the presence of significant amounts of hydroxy- or water-containing calcium phosphate phases [54–55].

FTIR spectroscopy (Figure 1A) further corroborates the formation of calcium phosphate. Products prepared with GAA after 24 h (CP^GAA24^) exhibit major bands for the phosphate group at 1022 and 1126 cm^−1^ (P–O ν_3_), 565 cm^−1^ (P–O ν_4_), and 990 cm^−1^ (P–O ν_1_) which can be attributed to the presence of PO_4_^3−^ and/or HPO_4_^2−^ groups. The IR spectra of the calcium phosphates precipitated in the presence of glacial acetic acid (GAA) after 48 h (CP^GAA48^) mainly shows bands associated with apatite at 1045 and 1169 cm^−1^ (P–O ν_3_), 563 and 606 cm^−1^ (P–O ν_4_ ), and 960 and 802 cm^−1^ (P–O ν_1_).

Similar spectra were observed for the samples precipitated in the presence of NaOH after 24 and 48 h (CP^NaOH24^ and CP^NaOH48^). Here the bands are at 1034 and 1168 (P–O ν_3_), 563 and 602 (P–O ν_4_), and 963 and 802 cm^−1^ (P–O ν_1_). This suggests that the CP^GAA48^, CP^NaOH24^, and CP^NaOH48^ materials are structurally similar.

X-ray diffraction (XRD, Figure 1B) shows that the addition of glacial acetic acid (GAA) or NaOH, respectively, to the reaction mixture leads to different calcium phosphates. XRD patterns of CP^GAA24^ show reflections at 2θ (°) = 13.08, 26.66, 28.52, 30.14, 32.70, 35.86, 40.16, 45.54, 47.52, 49.24, 50.88, 53.08, and 54.64, which can be assigned to monetite (CaHPO_4_, dicalcium phosphate anhydrate, DCPA, ICDD09-0080). In contrast, XRD patterns of CP^GAA48^ show intense reflections at 2θ (°) = 26.18, 28.46, 32.20, 34.00, 39.10, 46.36, 49.48, 53.70, 62.16, and 63.86. They can be assigned to either hydroxyapatite (Ca_5_(OH)(PO_4_)_3_, HAP, ICDD01-1008) or chlorapatite (Ca_5_(Cl)(PO_4_)_3_, ClAP, ICDD33-0271, ICDD24-0214) but the experimental data match better with ClAP.

The addition of NaOH instead of GAA leads to the formation of HAP or ClAP already after 24 h (CP^NaOH24^); an exact assignment of the reflections at 2θ (°) = 26.02, 28.42, 31.94, ca. 46.6, 49.64, 53.49, and 63.90 is difficult due to the fact that the reflections are very broad and the reflections of HAP and ClAP are very close.

Reflections in the XRD patterns of CP^NaOH48^ at 2θ (°) = 26.14, 28.42, 31.78, ca. 46.88, ca. 49.40, and 53.48 can again be assigned to HAP or ClAP. As the reflections are broader than in the patterns obtained from samples grown with GAA and because the positions of the reflections in HAP and ClAP are very close, it is difficult to make an irrefutable assignment to either HAP or ClAP. No indication of brushite or monetite can however be observed here.

**Figure 2:**
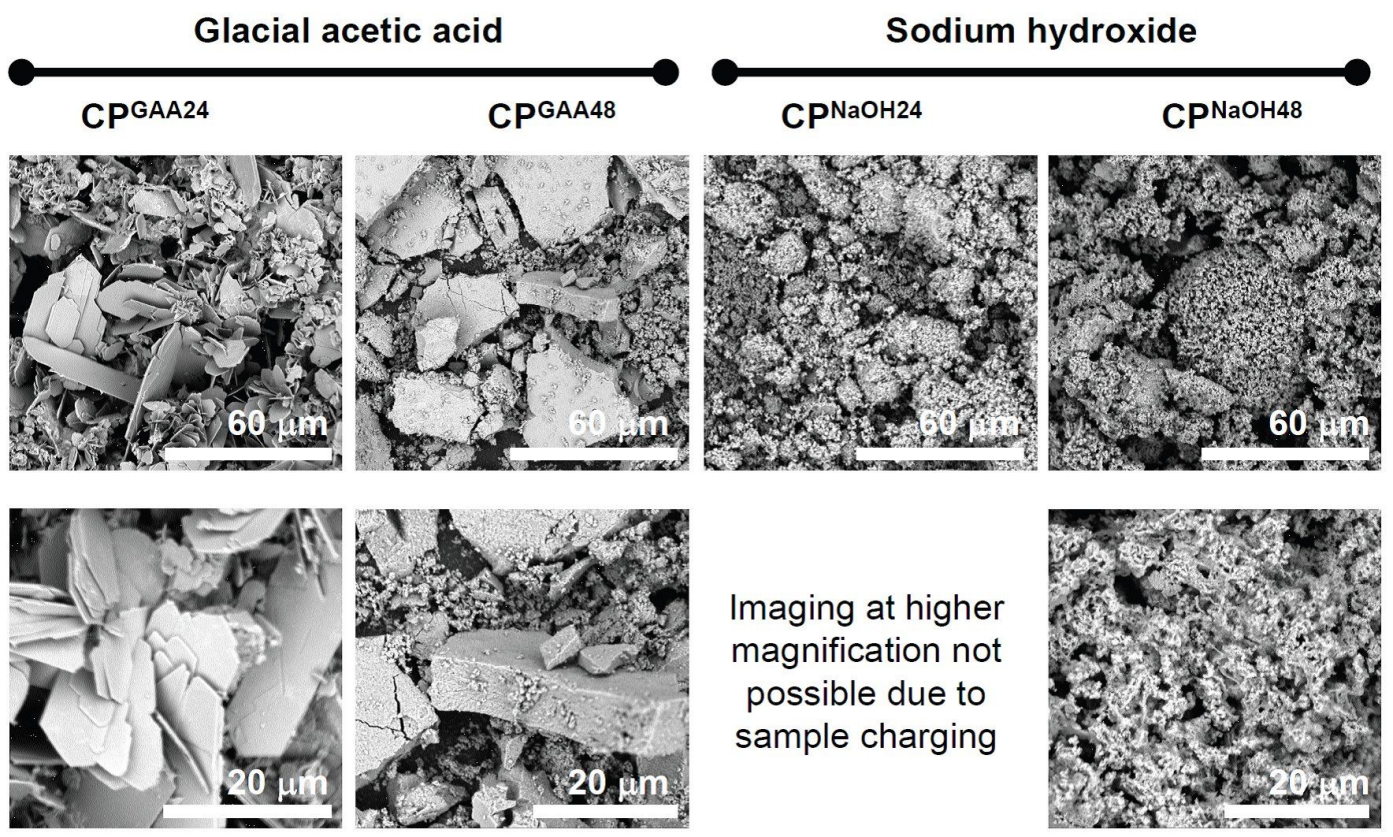
Low magnification (top row) and higher magnification (bottom row) SEM images of the precipitates. High magnification imaging of CP^NaOH24^ led to rapid sample charging and very poor imaging conditions even after sputtering; no image is thus shown.

Figure 2 shows representative scanning electron microscopy (SEM) images of the precipitates. With addition of GAA, SEM shows a clear morphological transition between the samples isolated after 24 and 48 h of reaction time, consistent with IR and XRD data. At 24 h, large and thin platelets form. Their size distribution is broad (from ca. 2 to 80 μm) and the crystal shapes are well developed. In most cases the plates are not present as individual platy crystals, but they form dense aggregates and exhibit steps and overgrowth of other crystals. At 48 h, the samples are dense large blocks with thicknesses in the micrometer range. These large blocky features are accompanied by smaller, less densely aggregated nanoparticles with sizes in the 100 to 300 nm range. These particles form small irregular aggregates with diameters of a few micrometers. All features (the large blocks and the less dense aggregates) are composed of smaller nanoparticles in the 100 nm range. These particles appear to be the primary constituents of all larger features observed in the SEM.

In contrast to the samples grown with GAA, the samples grown with NaOH exhibit a relatively uniform morphology, where small particles with sizes of 100 to 200 nm aggregate into larger structures. The main difference between the samples isolated at 24 and 48 h is the increased aggregation of the smaller particles. That is, at longer reaction times, the aggregated features are larger and reach tens of microns at 48 h. Moreover, the reaction in the presence of NaOH appears to favor an open structure with interstitial spaces with a few 100 nm to a few microns in diameter. Overall, the sample morphologies of the powders obtained in the presence of NaOH is more uniform than in the samples obtained in the presence of GAA.

Table 2 summarizes data obtained from energy dispersive X-ray spectroscopy (EDXS). The samples grown in the presence of GAA have a Ca/P ratio of 0.8 to 0.9. This is on the order of the Ca/P ratio of 1 in stoichiometric DCPD or DCPA [12–13,56]. Moreover, CP^GAA24^ also contains roughly equivalent amounts of sodium and chlorine, while the amount of chlorine in CP^GAA48^ is higher at around 2.3%. Consequently, the Ca/Cl ratios are slightly different at 8.6 and 6.7, respectively, at 24 and 48 hours of reaction.

**Table 2:** EDXS data of the precipitates; n.d. = not detected. EDXS does not observe any nitrogen indicative of the ionic liquid. Elemental analysis (EA) finds ca. 1% of carbon. Nitrogen is below the detection limit of the EA instrument (0.3%).

Sample	Ca [atom %]	P [atom %]	Cl [atom %]	Na [atom %]	N^a^ [atom %]	Ca/P	Ca/Cl

CP^GAA24^	15.6 ± 1.5	18.4 ± 0.8	1.8 ± 0.8	1.7± 0.1	n.d.	0.8 ± 0.1	8.6 ± 1.8
CP^GAA48^	15.4 ± 0.8	18.3 ± 0.2	2.3 ± 0.1	1.3 ± 0.2	n.d.	0.9 ± 0.1	6.7 ± 2.1
CP^NaOH24^	21.6 ± 1.6	16.1 ± 0.9	1.1 ± 0.2	n.d.	n.d.	1.3 ± 0.2	19.7 ± 3.3
CP^NaOH48^	22.0 ± 0.7	15.8 ± 0.1	1.2 ± 0.6	n.d.	n.d.	1.4 ± 0.1	18.3 ± 3.4

^a^From elemental analysis.

Samples grown in the presence of NaOH have a Ca/P ratio of 1.3 to 1.4, which is typical (although at the low end [12–13,56]) for calcium-deficient HAP or ClAP. None of the samples grown with NaOH contains Na in measurable amounts, while the fraction of Cl is on the order of 1%. Consequently, the Ca/Cl ratio is much higher than in the samples grown with GAA.

### Cellulose/calcium phosphate hybrid materials

The neat cellulose used in this study is a white powder. During mineralization, the precipitation of the hybrid materials can be observed visually by the appearance of a solid in the IL. The cellulose/calcium phosphate hybrid (CCPH) materials obtained after mineralization are either white (when synthesized in the presence of NaOH, see experimental part) or light brown (when synthesized in the presence of glacial acetic acid, GAA). This color change may be due to the acid-induced degradation of the cellulose by HCl [37,57–58] produced during the mineralization reaction: The reaction of the CaCl_2_ and NaH_2_PO_4_ yields the desired calcium phosphate precipitate along with NaCl and HCl as side products. While NaOH is able to neutralize some of the HCl formed during the reaction (and thus effectively removes acidic protons from the reaction mixture), GAA will contribute additional protons. The higher amount of protons in the latter case will then lead to a somewhat stronger acid-induced degradation of the cellulose. Alternatively, other degradation reactions of cellulose in ILs have also been reported [59–60]; these could also play a role here. Table 3 summarizes the reaction conditions.

**Table 3:** Reaction conditions for preparation of cellulose calcium phosphate hybrids (CCPH).

Sample	Additive	Reaction time [h]	Cellulose in IL [wt %]

Neat cellulose	—	—	—
CCPH1	GAA	24	3
CCPH2	GAA	48	3
CCPH3	GAA	24	6
CCPH4	GAA	24	9
CCPH5	NaOH	24	3
CCPH6	NaOH	48	3
CCPH7	NaOH	24	6
CCPH8	NaOH	24	9

**Figure 3:**
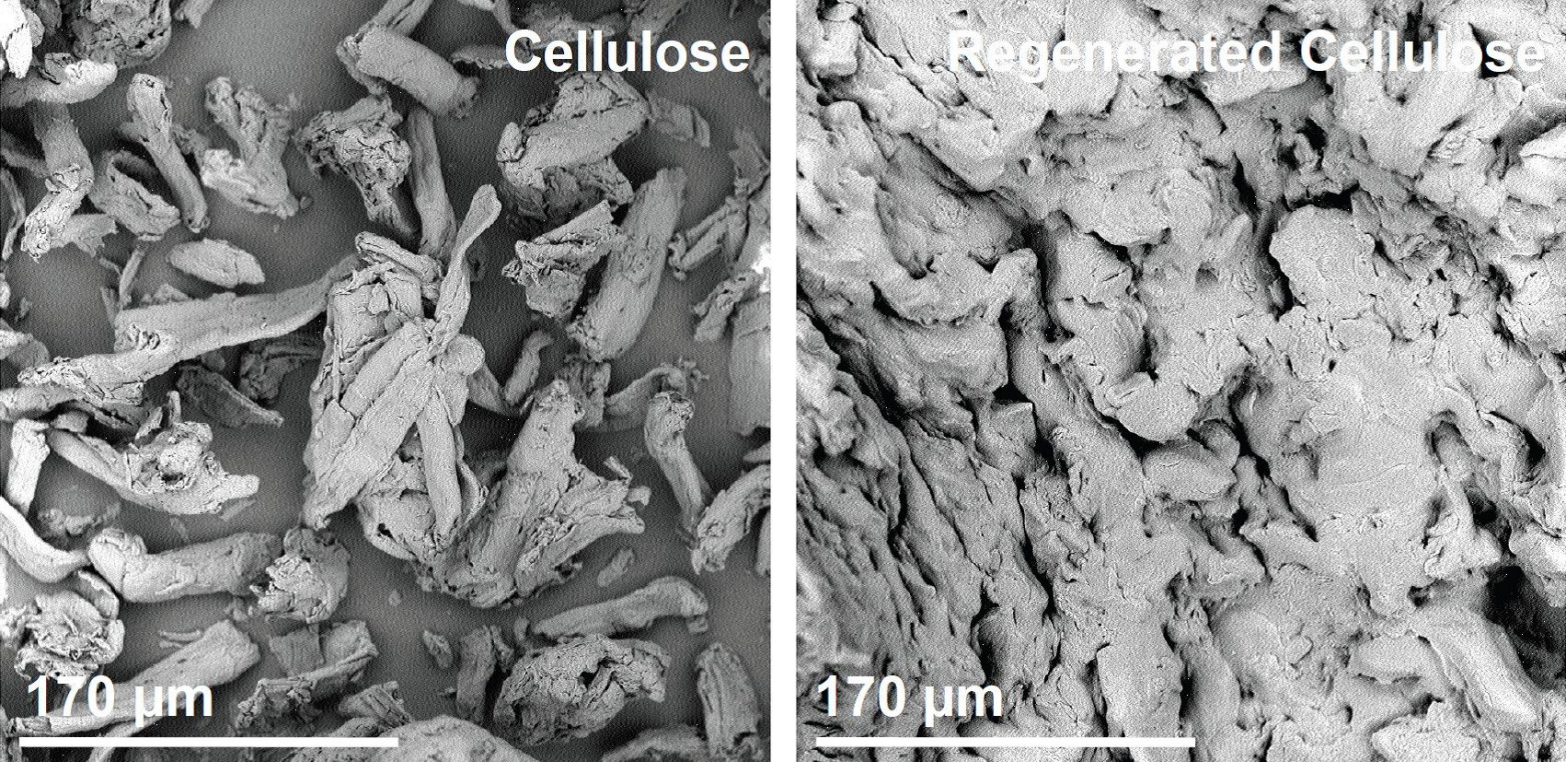
SEM images of as-received microcrystalline and regenerated cellulose.

Figure 3 shows representative SEM images of the as-received microcrystalline cellulose and cellulose regenerated from the IL 1-butyl-3-methylimidazolium chloride, [Bmim][Cl]. Neat, untreated microcrystalline cellulose consists of heterogeneous and highly aggregated fibers with sizes in the micrometer to hundreds of micrometers range. Regenerated cellulose exhibits a more uniform, less aggregated morphology of intertwined fibers with diameters on the order of tens of microns. This is consistent with other observations on cellulose reconstituted from ILs [34,58,61].

**Figure 4:**
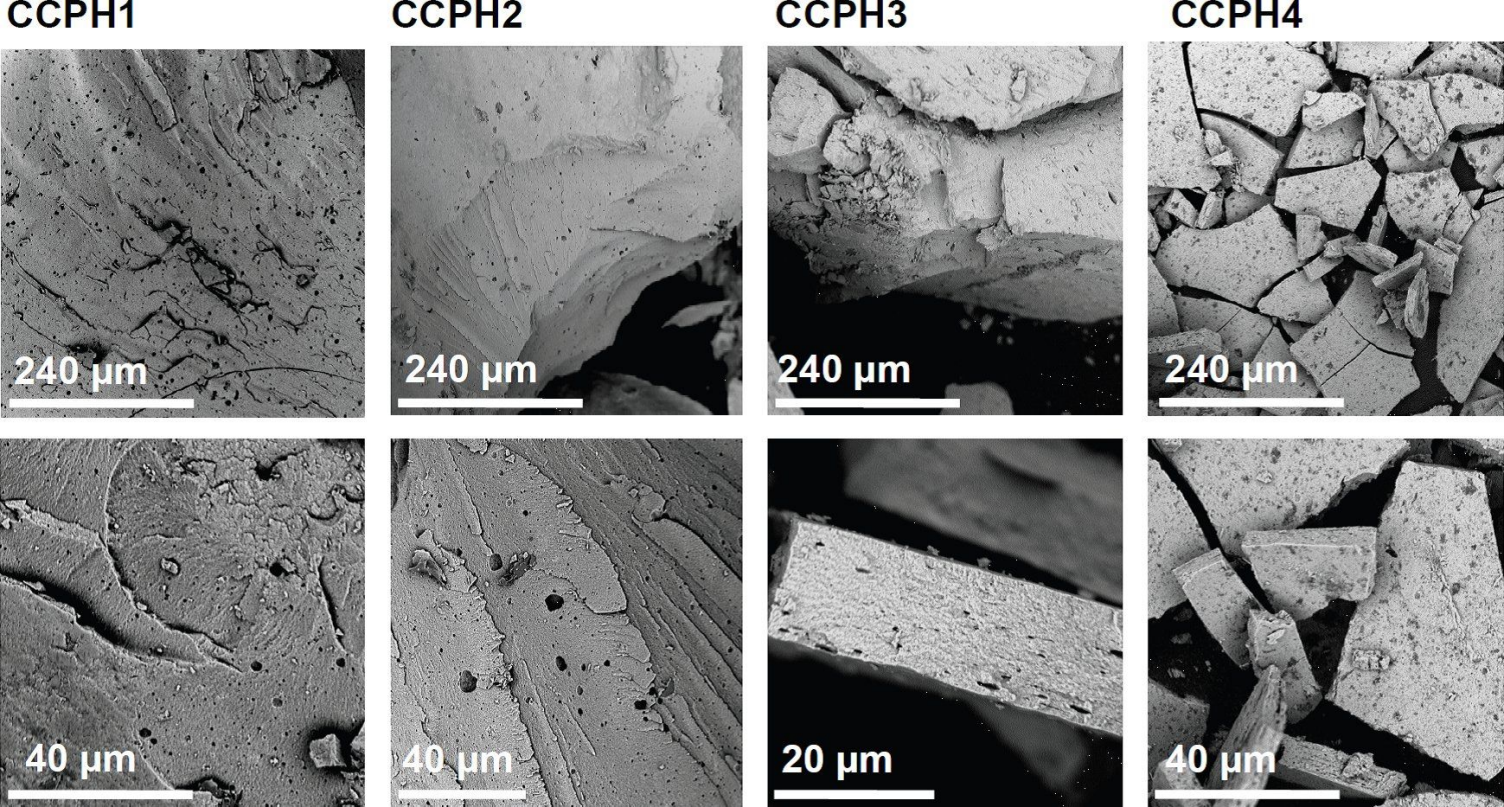
Low (top row) and high magnification (bottom row) SEM images of the hybrid materials prepared in the presence of GAA. Note the different scale bar in the lower row for CCPH3.

Figure 4 shows SEM images of the CCPH materials obtained after mineralization in the presence of GAA. SEM shows particles with sizes on the order of several hundreds of micrometers that are broken into pieces of several tens of microns with irregular shapes. All samples appear rather dense and no obvious pores can be observed. Moreover, closer inspection shows that the particles and fragments appear to have a layer-like architecture. Finally, the precipitates appear composed of small subunits, possibly of particles with diameters in the nanometer range, but this is, due to significant charging of the samples in the SEM, difficult to evaluate.

**Figure 5:**
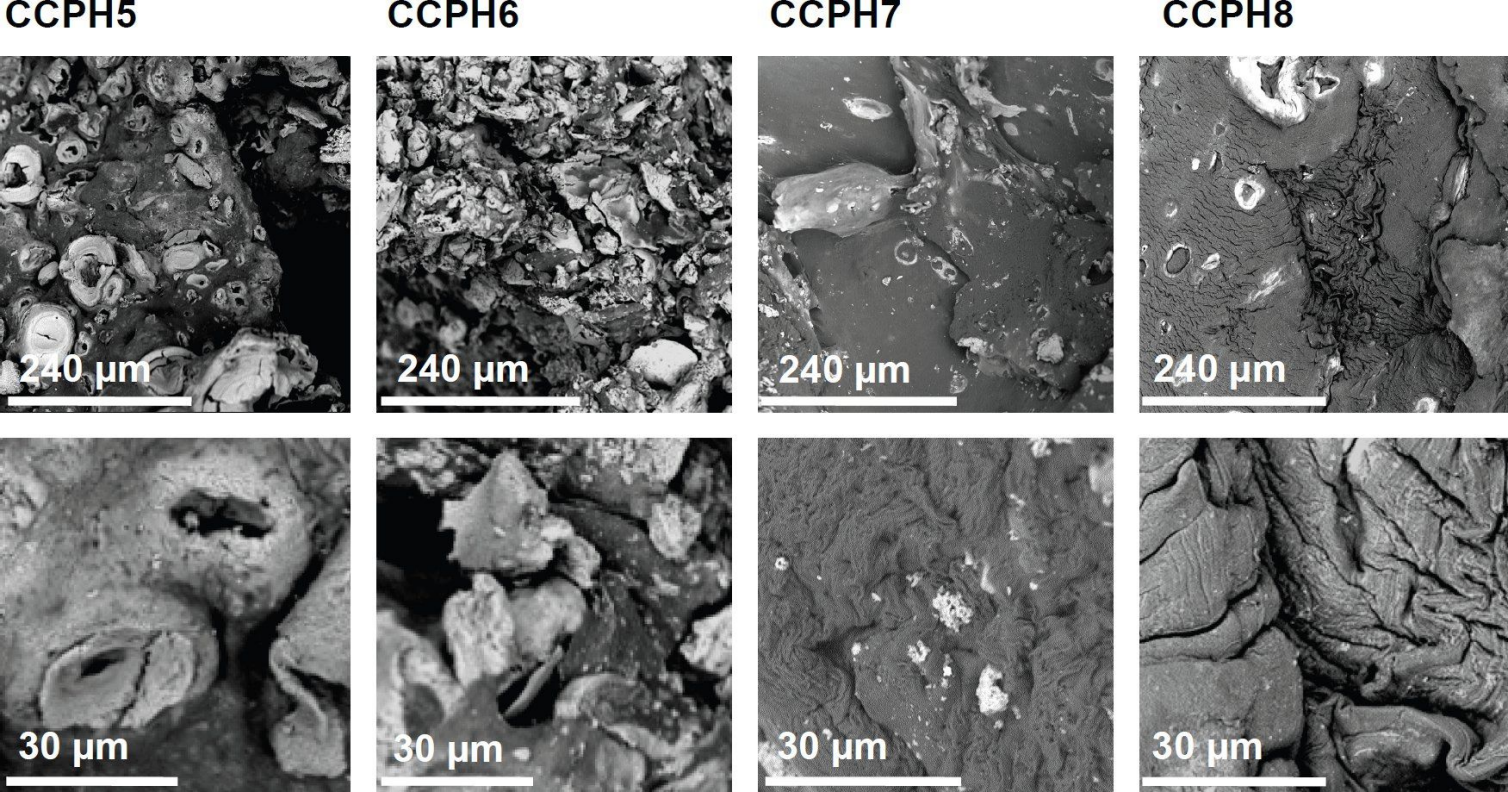
Low (top row) and high magnification (bottom row) SEM images of the hybrid materials prepared in the presence of NaOH.

Figure 5 shows that the addition of NaOH instead of GAA dramatically alters the product morphology. In contrast to GAA, the addition of NaOH leads to the formation of heterogeneous samples. At low cellulose concentrations (3%, CCPH5 and 6), the samples exhibit prominent round and holey features, presumably composed mostly of the inorganic, calcium phosphate. These features transform into smaller, poorly defined features after 48 h of reaction (CCPH6). The darker matrix material in these samples can be assigned to cellulose, because the brightness in the SEM images is roughly related to the atomic number of the respective region of the sample [62]. At higher cellulose concentrations, the round features are, although still present, much less prominent. In these samples, the morphology is largely defined by a darker background with morphologies similar to pure reconstituted cellulose, Figure 3.

**Figure 6:**
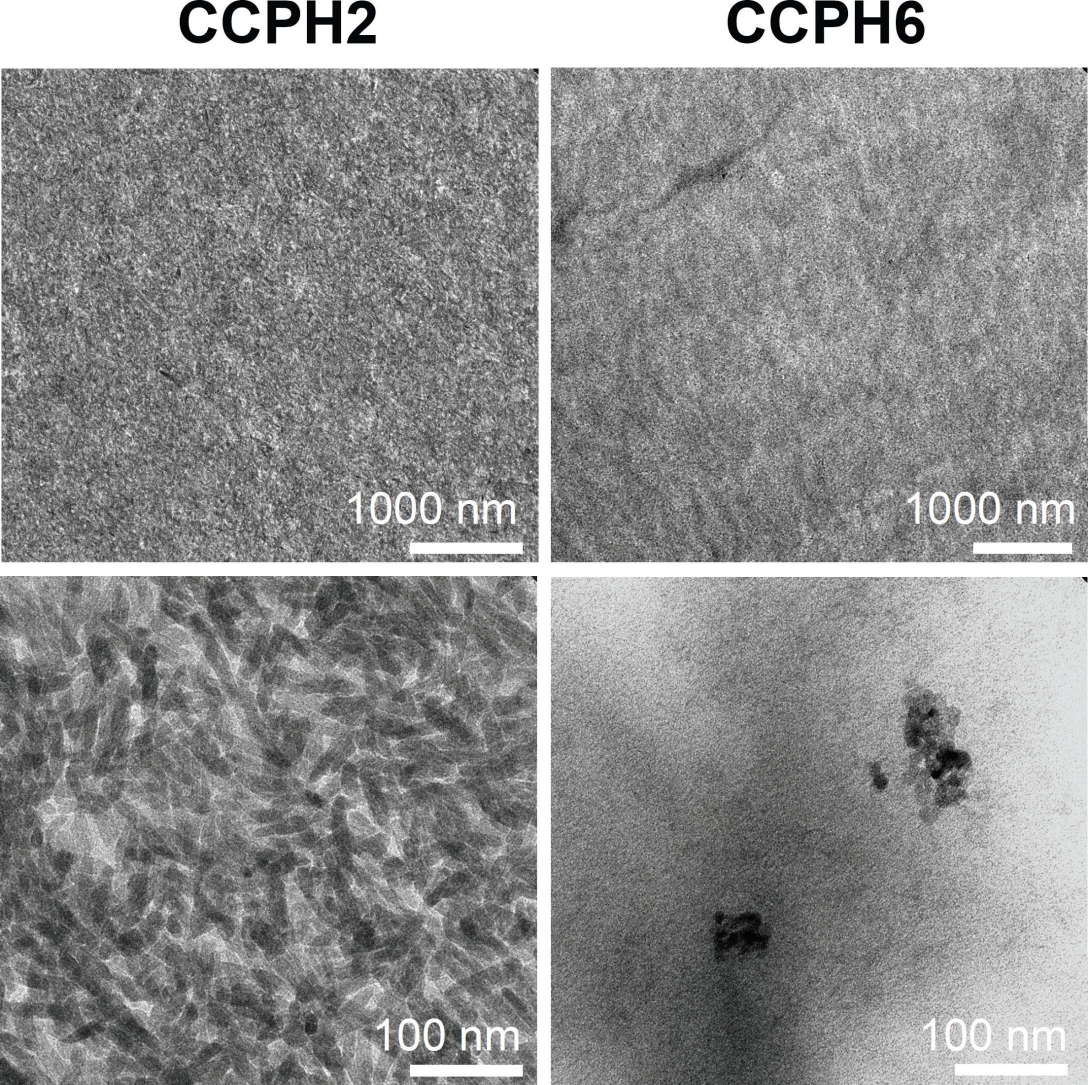
TEM images of thin sections of CCPH2 and CCPH6. Top row are low magnification and bottom row are high magnification images of the same samples.

Figure 6 shows representative TEM images of thin sections of GAA and NaOH. Overall, TEM cross-sections show a high conservation of structures between the two different approaches. Samples obtained in the presence of GAA (CCPH1-4) are highly homogeneous and consist of densely packed nanorods with a length on the order of 50–150 nm, that are densely packed, but, unlike a previous example [63], do not exhibit a common preferred orientation. In contrast, samples obtained with NaOH (CCPH5-8) appear homogeneous at lower magnifications, but higher magnification imaging clearly reveals their heterogeneous structure. The samples exhibit regions with low degrees of mineralization inorganic particles, sparsely mineralized regions and densely mineralized regions. The individual particles are roughly spherical and have a diameter of around 10–30 nm. Most particles are highly aggregated and form clusters of 100–200 nm in diameter; often also larger aggregates are observed.

Table 4 shows energy-dispersive X-ray spectroscopy (EDXS) data of the samples. The samples prepared with NaOH have Ca/P ratios between 1.2 and 1.3. This ratio is lower than the Ca/P ratio of 1.67 in pure stoichiometric hydroxyapatite (HAP) but Ca/P ratios lower than 1.67 are known for HAP and usually assigned to calcium-deficient apatite. Alternatively, the Ca/P ratios from EDXS could also indicate the formation of amorphous calcium phosphate (Ca/P = 1.5), octacalcium phosphate (OCP, Ca/P = 1.33), or β- or γ-tricalcium phosphate (TCP, Ca/P = 1.5) [13,56,64], or a mixture of phases.

**Table 4:** EDXS data of the CCPH materials.

Sample	Ca [atom %]	P [atom %]	Cl [atom %]	Ca/P	Ca/Cl

stoichiometric HAP	22.73	13.64	n/a	1.67	n/a
stoichiometric ClAP (OH completely substituted by Cl)	23.81	14.29	4.76	1.67	5.00
CCPH1	18.2 ± 1.3	17.8 ± 0.2	1.8 ± 1.2	1.0 ± 0.06	10.1 ± 4.4
CCPH2	18.2 ± 2.1	17.1 ± 1.1	2.5 ± 1.2	1.1 ± 0.2	7.3 ± 4.7
CCPH3	17.1 ± 2.9	18.1 ± 1.1	1.1 ± 0.2	0.9 ± 0.2	15.5 ± 5.4
CCPH4	16.6 ± 1.1	18.4 ± 0.2	1.3 ± 1.5	0.9 ± 0.1	12.7 ± 3.9
CCPH5	19.2 ± 0.4	16.6 ± 0.5	3.2 ± 1.7	1.2 ± 0.1	6.0 ± 1.4
CCPH6	19.4 ± 1.9	16.2 ± 0.4	3.5 ± 1.8	1.2 ± 0.2	5.6 ± 1.9
CCPH7	19.7 ± 0.5	16.7 ± 0.8	1.8 ± 1.6	1.2 ± 0.1	10.9 ± 1.9
CCPH8	20.4 ± 1.6	16.4 ± 0.8	1.5 ± 0.2	1.3 ± 0.2	13.6 ± 1.6

Samples grown in the presence of GAA have a Ca/P ratio of ca. 1 after 24 h of reaction time and ca. 1.2 after 48 h. The Ca/P ratio of ca. 1 is indicative of brushite or monetite, two calcium phosphate phases that precipitate (in aqueous media) at rather low pH. The ratio of 1.2 is rather unspecific and could indicate the formation of most of the above phases, although DCPD and DCPA are usually less prone to forming non-stoichiometric products than the other calcium phosphates.

EDXS also suggests that there are compositional differences between the samples. Generally, the chlorine content of the samples grown with higher cellulose content (CCPH3, 4, 7, 8) appears lower than the content of the samples grown at lower cellulose concentrations (CCPH1, 2, 5, 6). Moreover, EDXS seems to suggest that the chlorine content is slightly higher in the samples grown with NaOH (CCPH5, 6) instead of GAA (CCPH1, 2). These data must however be treated carefully because of their large errors: the standard deviations of most datasets are large and there is a significant overlap of the data; EDXS is thus not able to clearly distinguish between the different samples.

**Figure 7:**
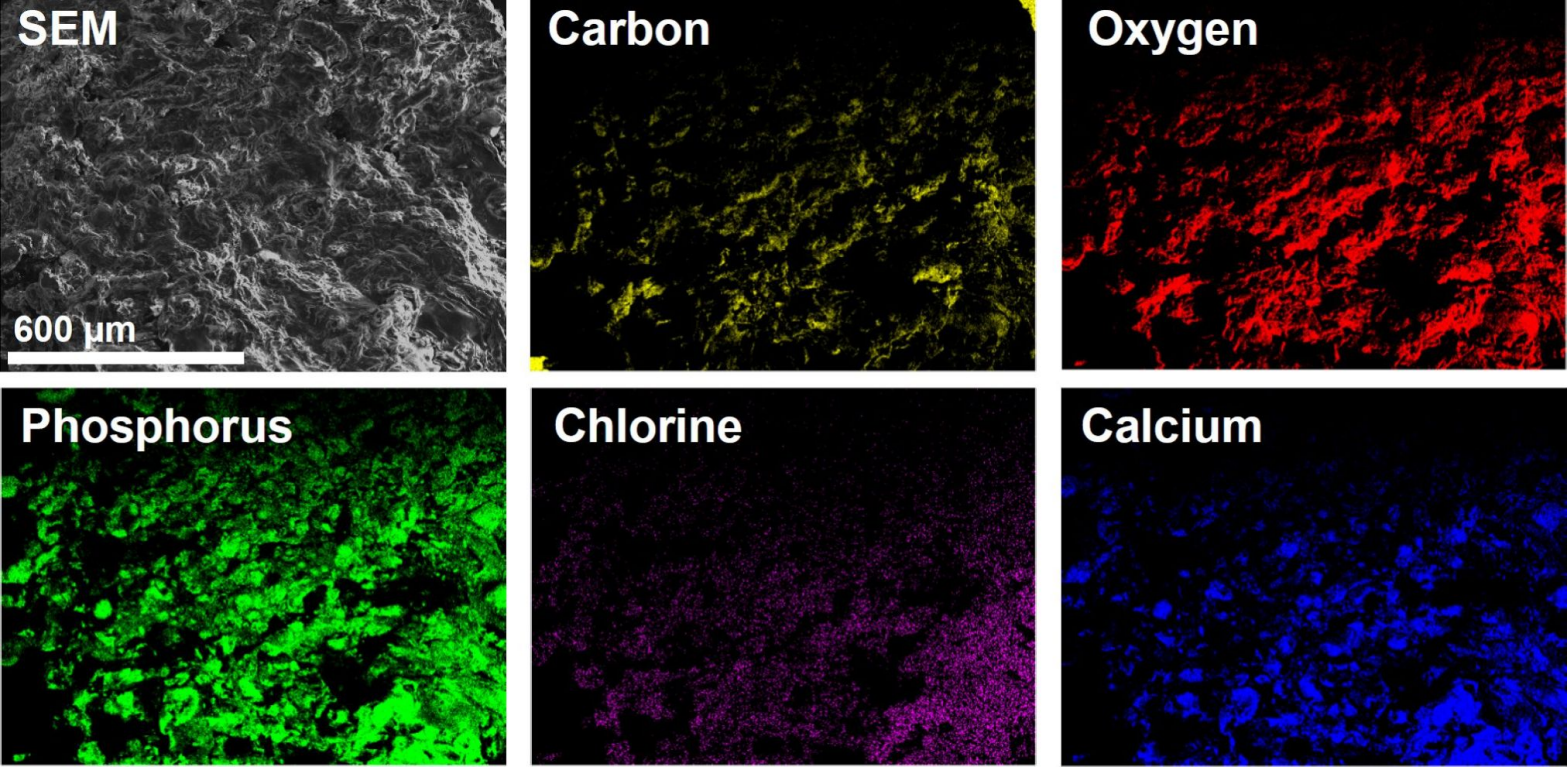
SEM image and elemental map of CCPH6.

Figure 7 shows representative X-ray elemental maps of all elements detected in energy dispersive X-ray spectroscopy (EDXS), that is, carbon, oxygen, phosphorus, chlorine, and calcium. The maps indicate fairly homogeneous elemental distributions on a hundreds of micrometers length scale even in CCPH5 to CCPH8, which suggests that all materials are uniform over the mm length scale. While carbon (from the cellulose), oxygen (from cellulose and calcium phosphate), phosphorus, and calcium (both from calcium phosphate) can be expected in these samples, the presence of chlorine and its homogeneous distribution throughout the sample is somewhat unexpected but highly reproducible. The fact that the location of the chlorine signal overlaps with the calcium and phosphorus signals suggests that it is also part of the mineral phase, possibly as chloride in chlorapatite.

**Figure 8:**
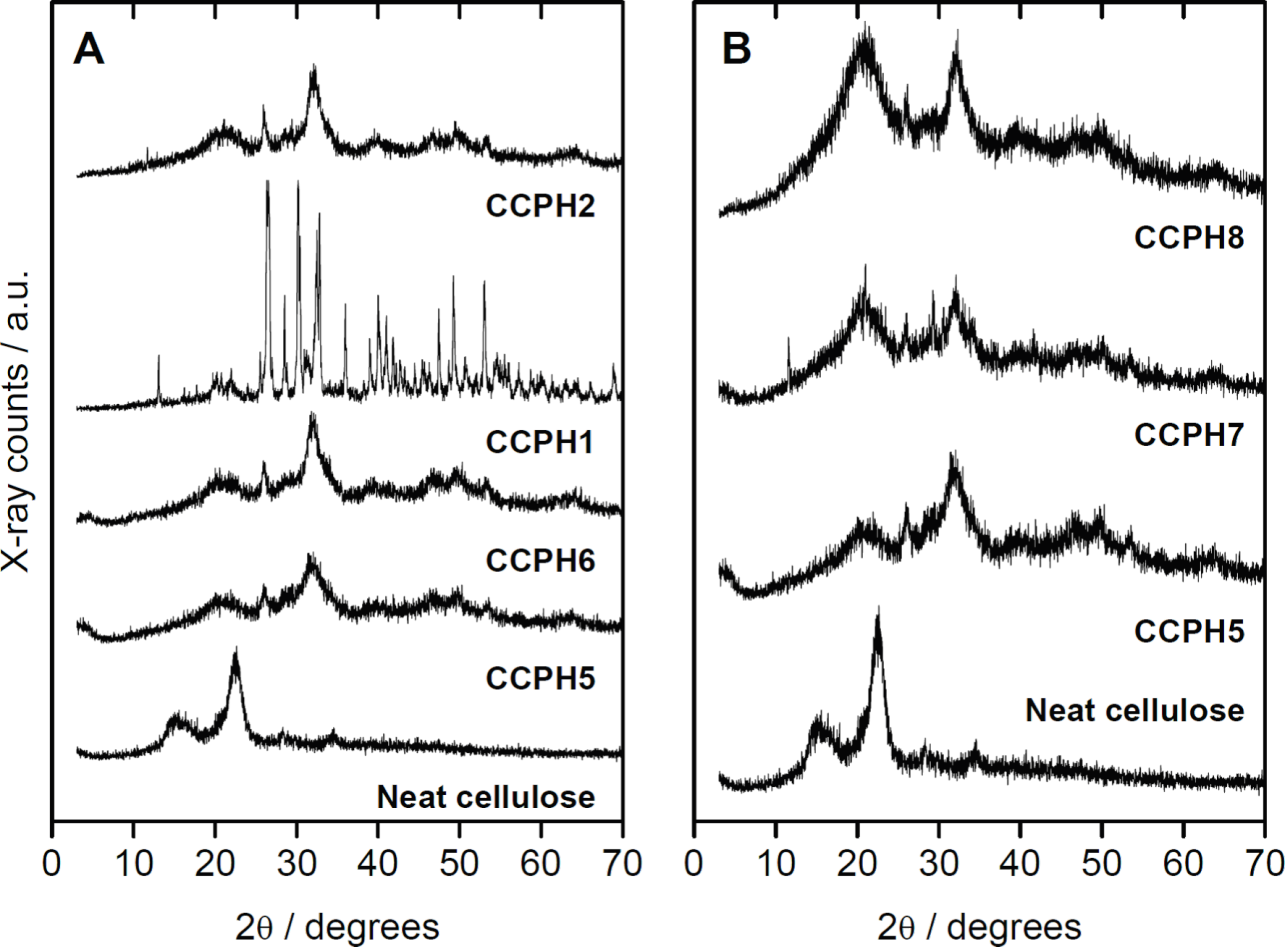
XRD patterns of cellulose and mineralized samples. Panel A shows effects of acid or base addition, panel B shows effects of cellulose concentration in the case of the samples grown with NaOH.

In spite of the limitations of the EDXS data just discussed, EDXS clearly shows, by way of the low Ca/P ratios, that the samples obtained by mineralization from the IL likely are crystallographically poorly defined. This is supported by powder X-ray diffraction (XRD), which in all cases yields patterns with broad reflections indicative of small crystallites, poor crystallinity, and poor crystallographic correlation, Figure 8.

Patterns of neat microcrystalline cellulose show reflections at 15.1 and 22.8° 2θ; these can be attributed to the crystalline structure of the cellulose. XRD patterns of all samples show that the order of the cellulose decreases after reconstitution from IL because the cellulose reflections are significantly broader after regeneration.

XRD patterns of samples prepared in the presence of GAA after 24 h show reflections at 2θ (°) = 13.1, 27.0, 30.5, 33.0 and 49.2, which can be assigned to monetite and, possibly, brushite. Patterns of samples obtained after 48 h show reflections at 2θ (°) = 26.0, 28.6, 32.3, 39.0, and 49.4, which can be assigned to HAP or ClAP. Similarly, samples prepared in the presence of NaOH after 24 and 48 h show broad reflections; they can again be assigned to HAP or ClAP. Increasing cellulose concentrations yield in all cases samples consisting of HAP and cellulose.

**Figure 9:**
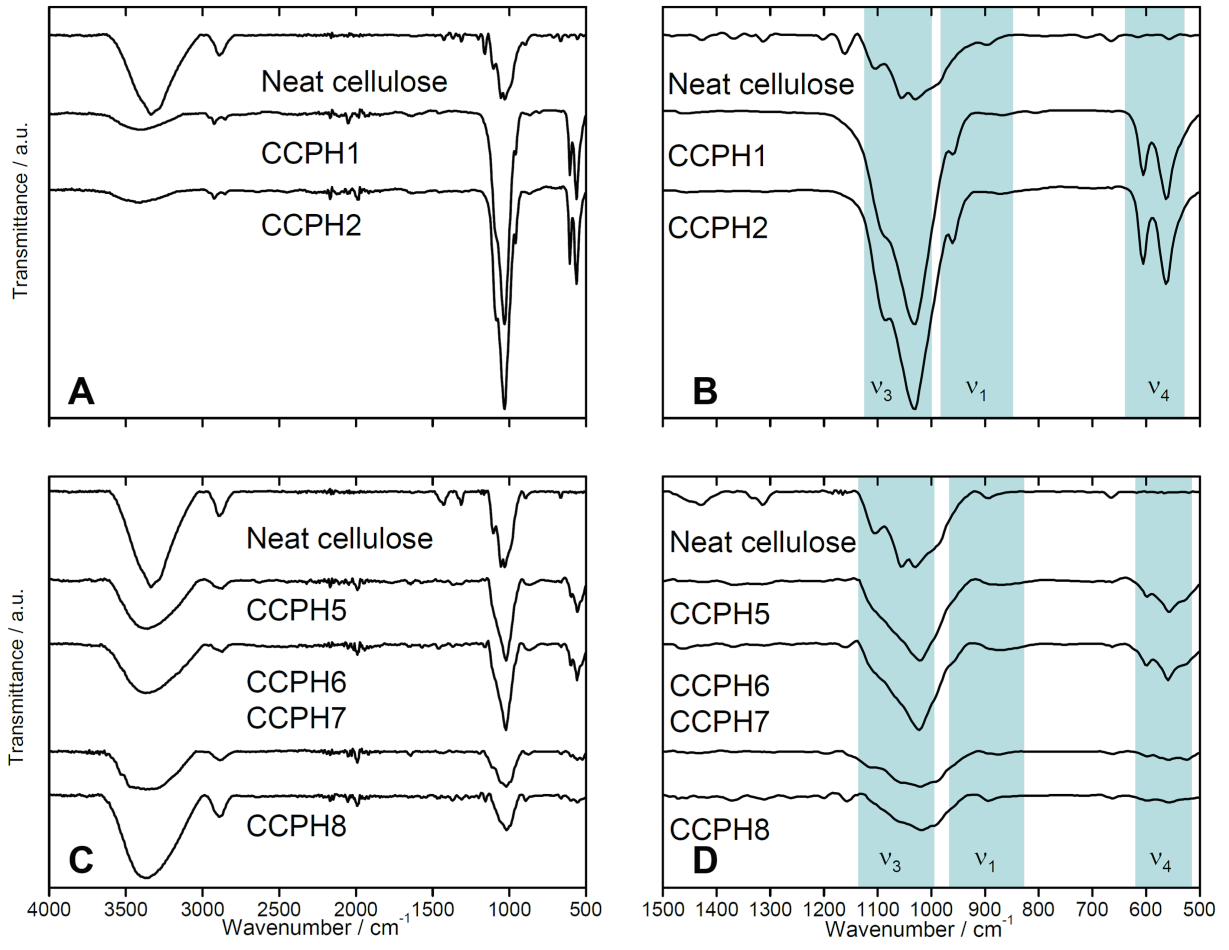
ATR-FTIR spectra of neat cellulose, for sample nomenclature see Table 3. Panels B and D are higher magnification views of the region showing the calcium phosphate vibration bands. Spectra are shifted vertically for better visibility.

Figure 9 shows representative attenuated total reflection-Fourier transform infrared (ATR-FTIR) spectra of the samples. Neat cellulose exhibits bands at 3335, 2890, 1427, and 1055 cm^−1^, which can be assigned to the OH, CH_2_, C–H symmetrical deformation, and C–O–C stretching vibration of cellulose, respectively.

The spectra of the hybrid materials prepared with GAA (CCPH1, 2, 3, and 4) show intense bands at 1031, 1090 cm^−1^ (P–O ν_3_), 562, 605 cm^−1^ (P–O ν_4_), and 956 cm^−1^ (P–O ν_1_), which can be attributed to the presence of PO_4_^3−^ and/or HPO_4_^2−^ groups. The intensity of the cellulose bands increases with increasing cellulose concentration from 3 to 6 to 9% of cellulose. The weak and broad –OH vibration band at around 3400 cm^−1^ in CCPH1 and CCPH2 is presumably due to the hydroxyl groups of cellulose, water, and hydroxide ions in the calcium phosphate. The relatively low intensity of the band suggests that (i) the fraction of cellulose is relatively low or that the –OH groups are strongly coordinated to the calcium phosphate and (ii) that the calcium phosphate is low in water or hydroxide content.

In contrast, IR spectra of the samples grown in the presence of NaOH at 3% cellulose concentration (CCPH5, 6) show typical bands associated with apatitic calcium phosphates. Bands at 960 (P–O ν_1_), 563 and 601 (P–O ν_4_), 1029 and 1095 cm^−1^ (P–O ν_3_) [54–55]. However, the calcium phosphate/cellulose hybrids prepared in the presence of NaOH at 6% and 9% of cellulose (CCPH7, 8) show slightly shifted and broadened phosphate bands at 901 (P–O ν_1_), 555 and 597 (P–O ν_4_), 1014 and 1160 cm^−1^ (P–O ν_3_). The intensity of the band at 3360 cm^−1^ suggests that (i) the fraction of cellulose is relatively high or (ii) that the calcium phosphate is relatively high in water or hydroxide content. Moreover, the spectra suggest, by way of the intense cellulose bands mentioned above, that, possibly, the degree of mineralization is lower than in the samples prepared in the presence of GAA. This is qualitatively supported by the fact that especially the intensity of the phosphate vibration bands is fairly low in the samples prepared at higher cellulose concentrations.

**Figure 10:**
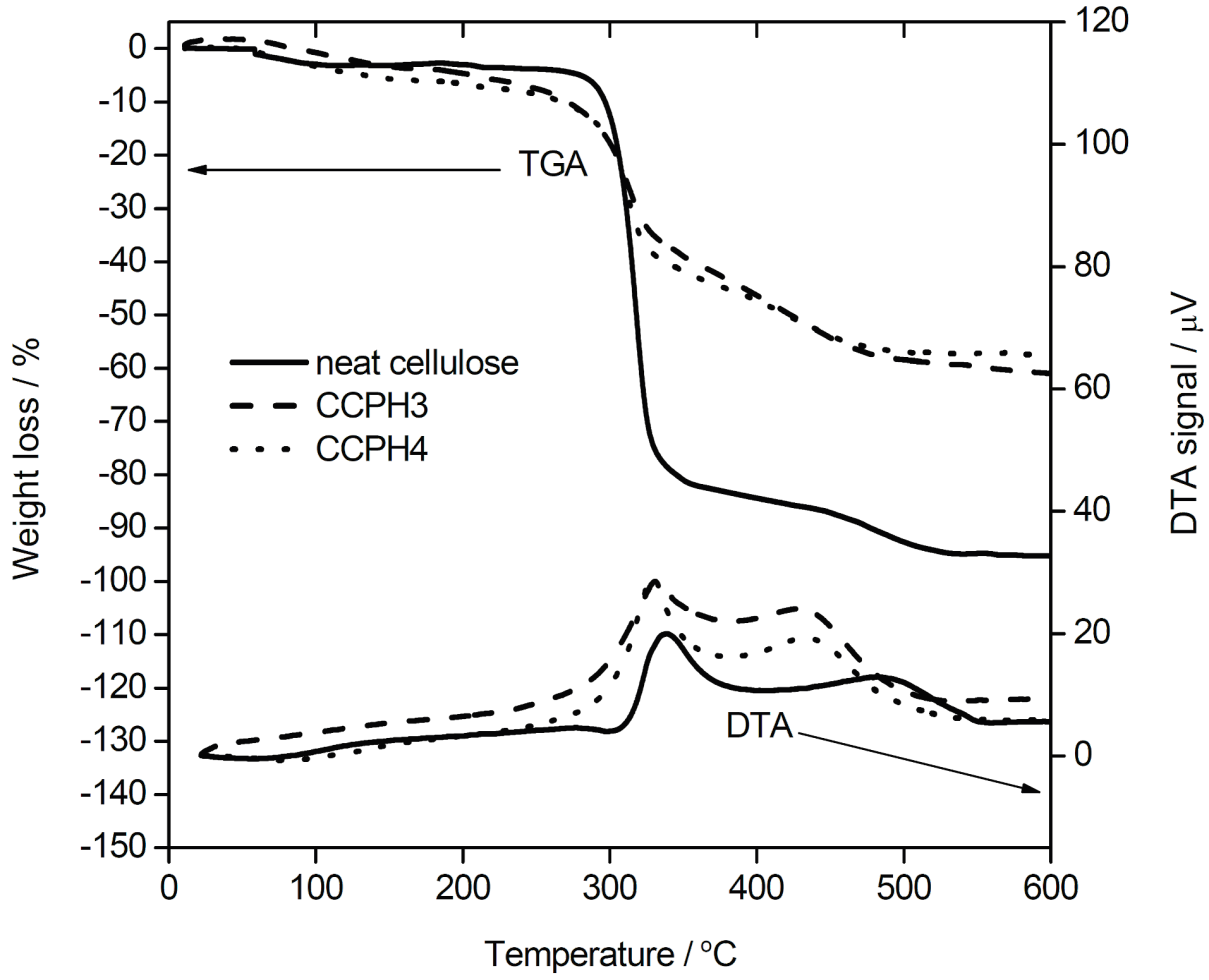
Representative TGA and DTA data of select samples. For full data see Table 3.

Figure 10 shows thermogravimetric analysis/differential thermal analysis (TGA/DTA) data. Table 5 summarizes the results from elemental analysis (CHN analysis) and TGA/DTA. TGA of the neat cellulose finds a weight loss of 96.1%, indicating that even the neat cellulose contains some fraction of non-volatile components. Overall the TGA curve is consistent with earlier data [65] on cellulose decomposition, where a first weight loss of ca. 4.5% is assigned to water desorption below ca. 120 °C. This initial weight loss is followed by the main decomposition step between ca. 280 and 340 °C (accounting for a loss of ca. 78.5%), followed by the final decomposition of the organic and carbonaceous residues up to 600 °C (13.1%). The corresponding DTA data confirms these assignments.

The same general observation can be made from the TGA/DTA data of all hybrid materials, Table 5. The samples exhibit a first weight loss of a few % assigned to water desorption and drying processes, followed by a two-step, thermally induced and exothermic, decomposition of the organic fraction. The fact that two steps are observed in TGA and two broad and overlapping, but distinct, signals in DTA clearly shows that the decomposition is in all cases a sequential but overlapping process.

Table 5 summarizes the results from TGA/DTA and elemental analysis (EA). EA shows that the carbon (= organic) content in the materials obtained in the presence of GAA (CCPH1, 2, 3, and 4) is lower than in the samples obtained with NaOH under the same conditions. This is supported by TGA, which also finds a lower overall weight loss in samples prepared with GAA (ca. 34–57%) than in samples prepared with NaOH (ca. 57–83%). Both EA and TGA therefore indicate that the mineralization in the presence of GAA is more effective in the sense that the fraction of inorganic is higher with the GAA additive than with NaOH. Both TGA and EA also show that, not surprisingly, the organic content in the hybrid materials increases as the initial cellulose concentration in the reaction mixture increases.

**Table 5:** EA and TGA data obtained for CCPHs. No N was detected in EA; the detection limit of the instrument is 0.3%.

Sample	C & H from EA [%]		Weight loss at 600 °C [%] (residue)	*T* of step in TGA [°C]			Theoretical C & H from TGA^a^ [%]	
	C	H		Step 1	Step 2	Step 3	C	H

Cellulose	43.18 ± 0.07	6.31 ± 0.0	96.01 (3.99)	246	350	530	42.70	5.92
CCPH1	11.59 ± 0.09	1.98 ± 0.08	34.71 (65.29)	218	338	597	13.04	2.06
CCPH2	21.80 ± 0.30	4.10 ± 0.10	56.49 (43.51)	218	343	596	21.90	3.35
CCPH3	24.46 ± 0.02	3.98 ± 0.04	59.60 (40.40)	182	333	462	21.00	3.50
CCPH4	26.90 ± 0.10	5.00 ± 0.10	69.30 (30.70)	183	342	458	25.10	4.20
CCPH5	23.49 ± 0.10	3.99 ± 0.14	62.91 (37.09)	167	340	597	24.53	3.73
CCPH6	23.00 ± 0.04	3.92 ± 0.02	57.36 (42.64)	215	368	590	21.26	3.39
CCPH7	29.19 ± 0.04	4.66 ± 0.04	75.98 (24.02)	249	348	596	27.89	4.50
CCPH8	32.84 ± 0.23	4.79 ± 0.33	82.71 (17.29)	252	342	593	31.94	4.89

^a^Theoretical amounts C and H were calculated from the fraction of organic material (= cellulose) as determined from TGA. The weight fraction of water (represented by the first weight loss at around 100 °C) was was subtracted and the molecular weight of anhydroglucose was used for calculation.

As stated in the introduction, calcium phosphate cellulose hybrid materials could be interesting biomaterials. Preliminary attempts to study the biocompatibility with MC3T3-E1 pre-osteoblasts, however, only provided qualitative information because of sample disintegration in the cultivation medium (PBS buffer). Likely this is due to the fact that the materials are quite brittle and tend to rapidly form a powdery product, which is difficult to handle quantitatively in cell assays. In spite of this, qualitative analysis showed that the pre-osteoblasts did proliferate on the hybrid materials. More detailed experiments are underway.

## Discussion

As stated in the introduction, ILs are interesting reaction media for the synthesis of advanced inorganic materials. ILs have, however, not been explored for the synthesis of inorganic biomaterials such as calcium phosphate, possibly for toxicity concerns [66–67]. The only examples the authors are currently aware of is an interesting study by de Zea Bermudez and colleagues, who have reported strong effects on the morphology of calcium carbonate but, interestingly, not on the crystal phase [53].

The current study shows that in all cases investigated here, calcium phosphate can be obtained from [Bmim][Cl]. In analogy to water-based precipitation reactions [12–13,68–69], the addition of an acid, GAA, or a base, NaOH, leads to different crystal phases (likely, one parameter that is significant here, is the presence of water traces). In the presence of GAA, DCPA forms, as can be verified from XRD (Figure 1B). XRD is further supported by FTIR spectroscopy (Figure 1A) which finds no –OH band at 24 h, indicating the formation of DCPA rather than DCPD, consistent with XRD. EDXS (Table 2) further supports these findings as it detects a Ca/P ratio of just below 1. Moreover EDXS also suggests that some sodium and chlorine are present in the samples grown with GAA. Although XRD does not show any indication of NaCl, we have previously observed the formation of minor NaCl fraction in a different system [70]. The formation of NaCl could thus also be possible here, especially because alkali salts are generally poorly soluble in ILs [65]. The low fraction of sodium and chlorine observed in the EDXS data could be due to the fact that the precipitates were washed with water after synthesis and most NaCl would thus have been washed out.

Additionally, both Na^+^ and Cl^−^ can also substitute into calcium phosphate; the residual fraction observed in the EDXS could thus also be incorporated in the calcium phosphate crystal lattice, although this is most common in the apatites and not in DCPA formed with GAA [12–13,64,71–72].

Moreover, SEM (Figure 2) shows that the particle size of the crystals grown with GAA after 24 h is orders of magnitude larger than the size of the crystals obtained after 48 h. This is similar to work by Shkilnyy et al. [73] who have shown that calcium phosphate grown from aqueous solution in the presence of poly(ethylene imine) follows a precipitation-redissolution-repreciptation pathway before forming the final product, HAP nanoparticles with a diameter on the order of 5–10 nm. The current study thus suggests that at least some of the findings from water-based calcium phosphate mineralization studies may have analogies in IL-based precipitation processes; this matter is however still under debate and more work is necessary to understand and quantify the intricacies of precipitation of inorganic matter from ILs.

SEM also shows that the samples grown from GAA-containing ILs are large blocks consisting of nanoparticles with diameters on the order of 100–300 nm. This suggests that the resulting materials could be mesocrystals [74–75]. At the moment this is, however, difficult to asses because the samples are highly unstable under the electron beam during electron diffraction.

In contrast to the samples grown with GAA, samples grown with NaOH are more uniform and SEM (Figure 2) shows the typical nanoparticle morphology that is also observed for calcium phosphate grown from aqueous solution at basic conditions [12–13]. Also consistent with conventional processes in aqueous solutions, XRD and FTIR spectroscopy (Figure 1) show that these precipitates are HAP and ClAP. Likely the reason for ClAP formation is the fact that the IL [Bmim][Cl] contains a high amount of chloride. The formation of Ca-deficient HAP or ClAP is further confirmed by EDXS (Table 2).

Importantly, EA and EDXS find no nitrogen in the precipitates. This suggests that the fraction of IL in the final materials is low. This is important for reasons of toxicity, as outlined above: if the precipitates synthesized in the current study are to be used in implantation or toothpaste, toxic compounds such as ILs must of course be removed. Apparently the washing process used here is sufficient to remove most of the IL such that no nitrogen (that is, IL cation) can be detected.

As pointed out in the introduction, there is a need for viable, flexible, and robust protocols towards (nano- and mesostructured) carbohydrate/calcium phosphate hybrid materials with the potential for scale-up. While the organic modification of cellulose in ILs has yielded a large number of publications [35,37,40,57] the synthesis of carbohydrate/inorganic hybrid materials from ILs is still in its infancy. The current study therefore addresses the problem by exploiting the potential of [Bmim][Cl] to both dissolve cellulose in significant weight fractions and to yield nanoscale calcium phosphate precipitates. Besides, we have also explored the effects of additives, NaOH and GAA, in the reaction mixture on sample architecture, crystal phase, crystal organization, and sample homogeneity.

SEM (Figures 2, 4, 5) and TEM (Figure 6) show that the additive, GAA vs NaOH, has a dramatic influence on the sample morphology. In the presence of GAA very uniform and highly organized nanoscale hybrid materials are obtained. In contrast, the addition of NaOH leads to heterogeneous sample morphologies with a poorly defined architecture of the inorganic building blocks. EDXS (Table 4) shows that all samples have Ca/P ratios that are lower than expected for stoichiometric HAP, although the initial Ca/P ratio in the reaction mixture was 1.67. In some cases (CCPH1, 2) the ratios of around 1 are indicative of DCPD or DCPA. Moreover, X-ray maps of elemental distribution (Figure 7) confirm SEM by showing that samples precipitated with NaOH are not homogeneous on a micrometer to nanometer scale.

Overall, the homogeneity of the samples precipitated with GAA suggests that here (i) the reaction mixture is homogeneous and nucleation and growth occurs throughout the reaction mixture or (ii) that the GAA molecules act as growth modifiers, possibly by stabilizing intermediates or nuclei which would then again yield the uniform particles observed in the TEM. Indeed, citrate has been suggested as a strong growth modifier for calcium phosphate from aqueous solution [76]. In contrast, the heterogeneity of the samples obtained with NaOH could be due to solubility issues of NaOH in the IL; as ILs are known to only poorly solubilize alkali halides [65]. A similar argument may apply to the case here.

The presence of Cl in all samples is surprising at first, but can be assigned to the fact that the reaction is done in an environment rich in chloride, the IL [Bmim][Cl]. The incorporation of chloride into the precipitates is further confirmed by XRD (Figure 8) because the XRD patterns can – at least partly – be assigned to chlorapatite.

XRD and IR spectroscopy (Figure 9) further show that in the presence of GAA not HAP or ClAP forms initially, but dicalcium phosphate anhydrate (monetite CaHPO_4_, DCPA). This is interesting because DCPA is a calcium phosphate phase that (in water) forms at relatively low pH values of around 5 [64,68,71–72]. This suggests that at least some of the growth of calcium phosphate in ILs, such as the effects of pH in water vs the presence of protons or hydroxide ions in IL, could be similar, but this claim will need further investigation.

The seeming discrepancy between the observation, that in the current work DCPA forms instead DCPD (which would be expected in aqueous media), can be resolved by the fact that the materials investigated in the current study were synthesized at 80 °C. At this temperature, DCPA also forms in aqueous media [68].

This observation, however, points to an issue with the current system. While most carbohydrates are fairly stable against temperature, it may for other reasons be desirable to operate at lower temperatures. To achieve this, [Bmim][Cl] is, due to its high melting point, not well suited. Other ILs such as acetates or formates would likely be more suitable candidates.

TGA/DTA (Figure 10, Table 5) and IR spectroscopy (Figure 9) show that the mineralization of calcium phosphate in the presence of NaOH yields materials with significantly lower degrees of mineralization than in the presence of GAA. This is different from aqueous systems, where the solubility product of HAP (formed at high pH) is significantly lower than that of DCPA (formed at low pH). Here the current study shows that concepts known from mineralization of calcium phosphate in aqueous media cannot in all cases directly be transferred to ILs. While both in water and ILs, higher temperatures seem to favor the formation of DCPA over DCPD, the higher degree of mineralization is somewhat counterintuitive when drawing inspiration from aqueous media: the reason for the higher mineralization level of the samples grown in the presence of GAA could well be related to issues of solubility products of the respective calcium phosphates in [Bmim][Cl] and these could be significantly different than in aqueous solution.

Finally it is important to address the aspect of biocompatibility and cytotoxicity. ILs are nowadays (after an initial phase, where this aspect was completely ignored) regarded as moderately toxic. This is mostly due to the fact that (i) many ILs either have long alkyl tails on the cation or that (ii) some of the anions such as PF_6_^−^ can degrade and form, among others, hydrofluoric acid, which is toxic [65–66]. In spite of this, the current data show that the extraction process used for sample purification is suitable to produce materials free of IL: neither EA (Table 5) nor EDXS (which is less sensitive, Table 2 and Table 4) detected any nitrogen in the current samples. As nitrogen is only present in the IL cation, this indicates that no more imidazolium moieties are present in the final, purified materials. Indeed, preliminary tests with MC3T3-E1 cells show that they proliferate on our materials without significant damage. This thus shows that IL-based synthesis protocols are also viable for biomaterials development.

## Conclusion

The current study presents a new approach towards true carbohydrate/calcium phosphate hybrid materials with a highly ordered, uniform, and chemically well defined mesostructure. The study has three key findings: (i) the use of suitable ILs enables the synthesis of hybrid materials with carbohydrates that have so far not been accessible for the formation of true nanoscale architectures in hybrid materials research, (ii) the addition of an acid or a base dramatically affects the outcome of materials synthesis; these data also suggest that some, but by far not all, concepts of calcium phosphate growth known from aqueous media can be transferred to ILs. Much more work is however needed to understand the processes leading to the observed morphologies. (iii) Soxleth extraction with a suitable solvent is a viable method for producing essentially IL-free hybrid materials that could find use in hard tissue repair or other fields. Clearly, as stated throughout the discussion, there are numerous open questions both with respect to synthesis optimization and the fundamentals of materials formation from ILs. This article is but the start down this interesting and promising new avenue of materials research and development.
